# Effects of Cannabidiol and Delta-9-Tetrahydrocannabinol on Emotion, Cognition, and Attention: A Double-Blind, Placebo-Controlled, Randomized Experimental Trial in Healthy Volunteers

**DOI:** 10.3389/fpsyt.2020.576877

**Published:** 2020-11-13

**Authors:** Timo Woelfl, Cathrin Rohleder, Juliane K. Mueller, Bettina Lange, Anne Reuter, Anna Maria Schmidt, Dagmar Koethe, Martin Hellmich, F. Markus Leweke

**Affiliations:** ^1^Department of Psychiatry and Psychotherapy, Central Institute of Mental Health, Medical Faculty Mannheim, Heidelberg University, Mannheim, Germany; ^2^Youth Mental Health Team, Brain and Mind Centre, Central Clinical School, Faculty of Medicine and Health, The University of Sydney, Sydney, NSW, Australia; ^3^Department of Psychiatry, Psychosomatics and Psychotherapy, Goethe University Frankfurt, Frankfurt, Germany; ^4^Department of Psychosomatics Medicine and Psychotherapy, Central Institute of Mental Health, Medical Faculty Mannheim, Heidelberg University, Mannheim, Germany; ^5^Institute of Medical Statistics and Computational Biology, Faculty of Medicine and University Hospital Cologne, University of Cologne, Cologne, Germany

**Keywords:** cannabinoids, cannabis, tetrahydrocannabinol, cannabidiol, healthy subjects, model psychosis, rct

## Abstract

The two main phytocannabinoids—delta-9-tetrahydrocannabinol (THC) and cannabidiol (CBD)—have been extensively studied, and it has been shown that THC can induce transient psychosis. At the same time, CBD appears to have no psychotomimetic potential. On the contrary, emerging evidence for CBD's antipsychotic properties suggests that it may attenuate effects induced by THC. Thus, we investigated and compared the effects of THC and CBD administration on emotion, cognition, and attention as well as the impact of CBD pre-treatment on THC effects in healthy volunteers. We performed a placebo-controlled, double-blind, experimental trial (GEI-TCP II; ClinicalTrials.gov identifier: NCT02487381) with 60 healthy volunteers randomly allocated to four parallel intervention groups, receiving either placebo, 800 mg CBD, 20 mg THC, or both cannabinoids. Subjects underwent neuropsychological tests assessing working memory (Letter Number Sequencing test), cognitive processing speed (Digit Symbol Coding task), attention (d2 Test of Attention), and emotional state (adjective mood rating scale [EWL]). Administration of CBD alone did not influence the emotional state, cognitive performance, and attention. At the same time, THC affected two of six emotional categories—more precisely, the performance-related activity and extraversion—, reduced the cognitive processing speed and impaired the performance on the d2 Test of Attention. Interestingly, pre-treatment with CBD did not attenuate the effects induced by THC. These findings show that the acute intake of CBD itself has no effect *per se* in healthy volunteers and that a single dose of CBD prior to THC administration was insufficient to mitigate the detrimental impact of THC in the given setting. This is in support of a complex interaction between CBD and THC whose effects are not counterbalanced by CBD under all circumstances.

## Introduction

Due to its relaxing and psychotropic properties, cannabis has been used for centuries for recreational purposes (1). It represents the worldwide most frequently used illicit drug (2) for decades, today, and probably in the years to come. Over the last years, a growing public debate on the legalization of cannabis for medical and recreational use took place. While in most regions, discussions about risks and chances are still ongoing, some countries or states (e.g., Uruguay, Colorado) already permitted a retail market of cannabis.

Most users consume cannabis only on a sporadic basis with a modest risk of severe adverse effects. However, cannabis use can cause severe impairments such as morphological brain changes (3, 4), cannabis use disorder (CUD) (5), persisting cognitive (6), memory (7), and behavioral (8) deficits as well as an increased risk of developing psychotic disorders (9–11). In particular, adolescents, who represent the majority of recreational users (3), are highly vulnerable, as the neuronal maturation of the brain is not yet completed. Furthermore, it must be considered that the content of the psychotomimetic ingredient delta-9-tetrahydrocannabinol (THC), is steadily rising from averaged 3% in the 1960s to 20% nowadays in high potency varieties in e.g., the Netherlands (11, 12), also contributing to the increased rate of CUD (5).

Various studies have shown that acute THC administration can induce transient psychosis in healthy volunteers as well as cognitive impairments and electroencephalography patterns comparable to psychosis (11). For this reason, THC can be used to induce a *model psychosis*, a term introduced in 1932 by Kurt Beringer (13) for psychotic-like symptoms intentionally caused by psychotomimetic drugs.

On the other hand, the therapeutic effects of the non-psychotomimetic phytocannabinoid cannabidiol (CBD) become more apparent (14–17). Furthermore, it has been speculated that CBD is able to mitigate some effects of THC or nabilone, a synthetic THC analogon, as CBD reduced THC induced anxiety (18) and attenuated the impact of the psychotomimetic cannabinoids on binocular depth inversion (19), episodic memory (20), facial emotional recognition (21), and psychotic symptoms (22) in healthy volunteers. Besides, it has been reported that a high CBD content in smoked cannabis reduced self-reported psychotic symptoms (23, 24) as well as anxiety ratings (25), and attenuated episodic memory-impairing effects (25). However, CBD was unable to reduce cognitive processing speed and working memory deficits induced by THC (20). Furthermore, varying CBD concentrations in smoked cannabis did also not affect working memory and sustained attention (26) as well as psychotomimetic symptoms (25), electroencephalographic measures, and event-related potentials (26).

As this previous data suggest that CBD and THC interact on at least some cognitive domains and anxiety, we conducted a placebo-controlled trial investigating the effects of a single oral placebo, CBD or THC administration as well as THC effects after pre-treatment with CBD on cognitive processing speed, working memory, attention, and emotional states in healthy volunteers. Importantly, we stratified for functional catechol-o-methyltransferase (COMT) polymorphism to exclude the influence of different dopamine elimination rates, as it has already been shown that cannabis affects cognition (including memory performance) in a COMT genotype-dependent manner (27, 28).

## Materials and Methods

This double-blind, randomized, parallel-group, placebo-controlled experimental trial (GEI-TCP II; ClinicalTrials.gov identifier: NCT02487381) was approved by the Ethics Committee II of the Medical Faculty Mannheim, Heidelberg University, Germany and the German Federal Institute for Drugs and Medical Devices (BfArM). All subjects gave written informed consent.

**Figure 1:**
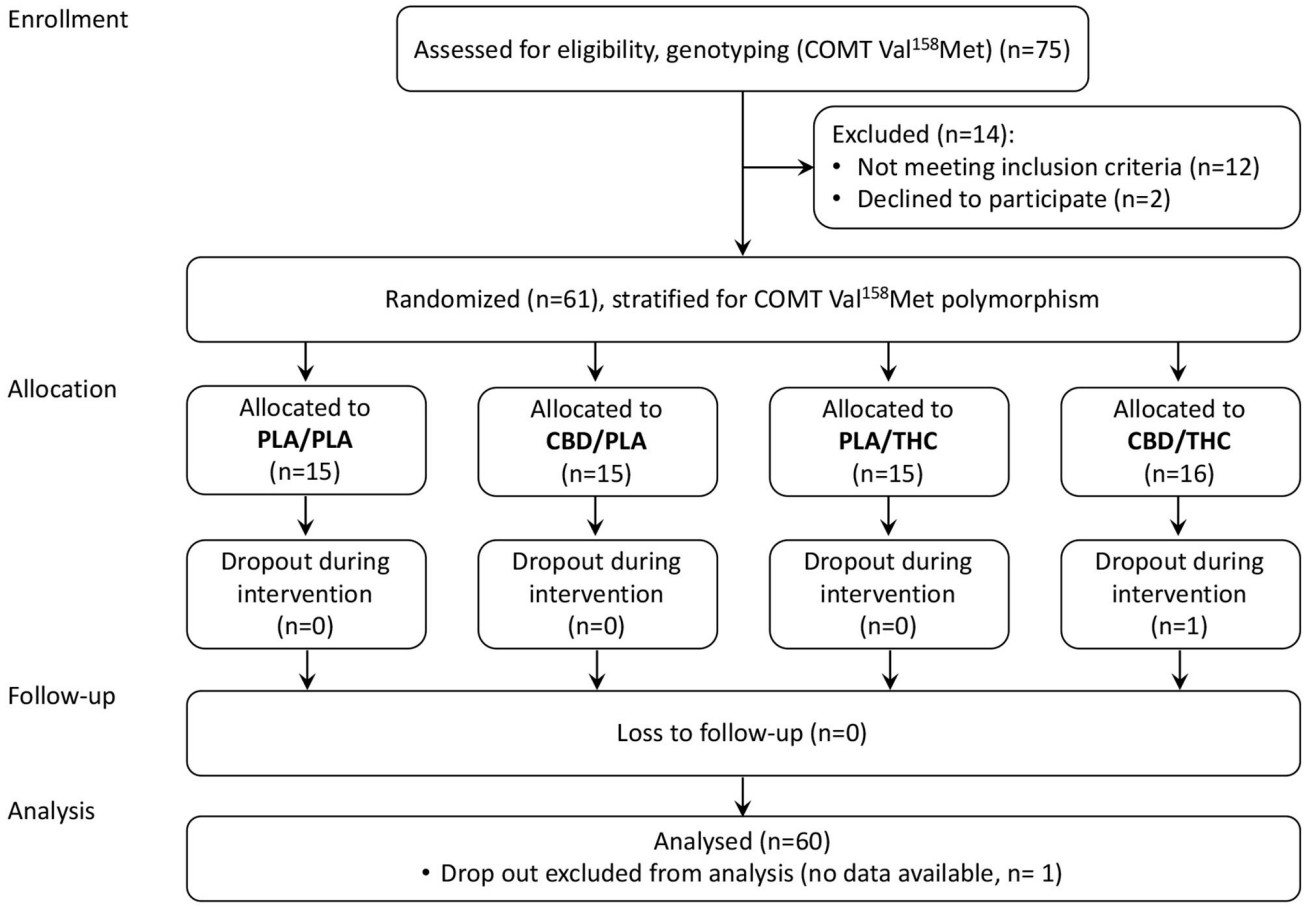
CONSORT Flow Diagram. CBD, cannabidiol; PLA, placebo; THC, delta-9-tetrahydrocannabinol.

As summarized in the CONSORT Flow Diagram (Figure 1), we screened 75 potential participants and enrolled 61 healthy volunteers. Sixty male, right-handed, participants aged between 19 and 36, completed the study per protocol. None of the subjects had any severe medical or neurological illness or personal or family history of psychiatric disorder. Subjects with a positive history of recurrent substance abuse, or cannabis consumption more than 10 times or less than one time during their lifespan, or cannabis consumption in the previous 6 months were excluded. All subjects had a body mass index (BMI) between 18 and 30 (kg/m^2^), a negative urine drug screening (including barbiturates, benzodiazepines, cannabinoids, methadone, opiates, amphetamine, ecstasy, and cocaine), and regular blood pathology results. Furthermore, no caffeine, alcohol, and nicotine consumption was allowed while participating in the trial. Volunteers were asked to abstain from these compounds. This study was limited to male volunteers because it was designed to parallel an investigation administering radionuclides.

Enrolled participants were stratified by their COMT Val^158^Met genotype to reduce the potential influence of different dopamine elimination rates and allocated to one of four parallel treatment groups, using computer-generated permuted blocks of varying length (allocation ratio 1:1:1:1).

### Experimental Procedures

Baseline assessments of all neuropsychological tests were carried out 1 day before the interventional day (V1).

During the interventional day (V2), all subjects had standardized breakfast and lunch. After breakfast, the first venous blood collection took place, and volunteers received either 4 × 200 mg CBD (>99.8% pure, STI Pharmaceuticals, Brentwood, UK), or corresponding placebo capsules. Thirty minutes later, administration of 2 × 10 mg THC (>98.8% pure, THC pharm, Frankfurt, Germany) or corresponding placebo capsules was scheduled. Approximately 205 min after CBD or placebo administration, blood withdrawal for THC level analysis by LC-MS MS (29) took place, followed by neuropsychological assessments.

For safety reasons, all subjects were clinically examined on the following day (V3), and all neuropsychological tests were repeated.

### Neuropsychological Assessments

All neuropsychological tests were carried out in paper-pencil versions.

To capture the participants' emotional state, we used a short version of the adjective mood rating scale (EWL) (30). This questionnaire comprises 60 adjectives and a four-point response format ranging from (0) not at all to (4) strongly. It covers the following six emotional categories: (a) performance-related activity, (b) general inactivation, (c) extroversion, (d) general well-being, (e) emotional excitability, and (f) depressiveness.

Cognitive processing speed was assessed using the Digit Symbol Coding task. Subjects were instructed to replace as many digits as possible in 90 s by given symbols, while the pairing table remained visible. Further, we evaluated working memory performance using the Letter-Number-Sequencing test. After reading an unordered sequence of numbers and letters aloud, subjects were asked to recall the numbers in ascending and the letters in alphabetical order. Both Digit Symbol Coding and Letter-Number-Sequencing are part of the Wechsler Adult Intelligence Scale (31).

In order to quantify changes in concentration and attention, the d2 Test of Attention (32) was used. During this paradigm, participants had to scan 14 test lines with 47 characters (“d” or “p” marked with a different number of dashes) and cross out all “d” characters marked with two dashed while ignoring all other characters. Volunteers were instructed to operate as fast as possible, but to minimize the error rate at the same time. The d2 Test of Attention provides multiple scores, of which we analyzed the following: error-corrected total number, and concentration capacity.

### Statistical Analysis

Statistical data analysis was performed using the software R (33).

The sample size of 15 subjects per treatment group, i.e., 60 subjects in total, is sufficient to detect a standardized difference of about 1.1 between any two groups (80% power, two-sided type I error 5%, two-sample Wilcoxon rank sum test, no multiplicity correction).

Group differences of continuous demographic variables were assessed using the Kruskal-Wallis test, while Fisher's exact test was used for the categorical variable “smoking.”

Intraindividual difference scores (investigational day—baseline) were calculated to evaluate the changes from baseline to the interventional day (~205 min post CBD intake) in emotional and attentional state and cognitive performance. The difference scores of the neuropsychological tests were not normally distributed. Thus, changes were analyzed by the non-parametric Kruskal-Wallis test, followed by closed testing, i.e., a stepwise multiple testing procedure that strongly controls the familywise error (34). The significance level was set at α ≤ 0.05 (two-sided). The data are presented as median (mdn) and 0, 25, 75, and 100 percentiles.

## Results

Sixty healthy volunteers participated in all sessions and completed the study per protocol. The four groups were adequately matched concerning age, BMI, cannabis lifetime use, intelligence, and smoking status (see Table 1).

**Table 1:** Demographics.

**Intervention-Groups**	**PLA/PLA**	**CBD/PLA**	**PLA/THC**	**CBD/THC**	**Statistical significance**
*N*	15	15	15	15	
COMT Val^158^ Met genotypes	5 Val/Val 5 Val/Met 5 Met/Met	5 Val/Val 5 Val/Met 5 Met/Met	5 Val/Val 5 Val/Met 5 Met/Met	5 Val/Val 5 Val/Met 5 Met/Met	
Age [years]	26 (21, 25, 28, 29)	25 (20, 24, 26, 37)	24 (20, 22, 26, 27)	27 (21, 23, 29, 33)	*p =* 0.226
BMI [kg/m^2^]	23 (20, 22, 25, 28)	26 (20, 22, 28, 30)	22 (20, 22, 25, 33)	23 (19, 22, 25, 29)	*p =* 0.340
Cannabis lifetime use	3 (1, 2, 4, 5)	6 (2, 4, 8, 10)	6 (6, 6, 6, 7)	5 (4, 4, 5, 6)	*p =* 0.540
Intelligence: MWTB	112 (92, 104, 118, 136)	110 (92, 101, 112, 124)	110 (89, 102, 112, 136)	112 (100, 104, 115, 130)	*p =* 0.734
Smokers N/Y	9/6	9/6	10/5	13/2	*p =* 0.294

*Data are presented as median (0, 25, 75, 100 percentiles) or number of subjects. BMI, body mass index; PLA, placebo; CBD, cannabidiol; THC, delta-9-tetrahydrocannabinol; COMT, catechol-o-methyltransferase; Val, valine; Met, methionine; MWT-B, Multiple Choice Intelligence Test*.

In general, individuals who received CBD plus placebo (CBD/PLA) did not show relevant differences compared to the placebo plus placebo (PLA/PLA) group, while THC following placebo (PLA/THC) administration significantly affected cognitive performance. In addition, THC induced more extensive changes in emotional states than PLA/PLA and CBD/PLA, although these alterations did not reach significance. Interestingly, pre-treatment with CBD did not attenuate THC effects in the CBD/THC group. CBD/THC subjects showed impairments of cognitive abilities well comparable to PLA/THC subjects. Furthermore, the observed changes in emotional states in CBD/THC subjects were comparable to those observed in PLA/THC subjects and significantly larger compared to CBD/PLA and PLA/PLA subjects.

### Effects of Exogenous Cannabinoids THC and CBD on Emotion

At the interventional day, treatment with phytocannabinoids changed subjects' self-evaluation of their emotional state in four of six categories. Ratings of general inactivation and general well-being did not differ between interventional groups.

**Figure 2:**
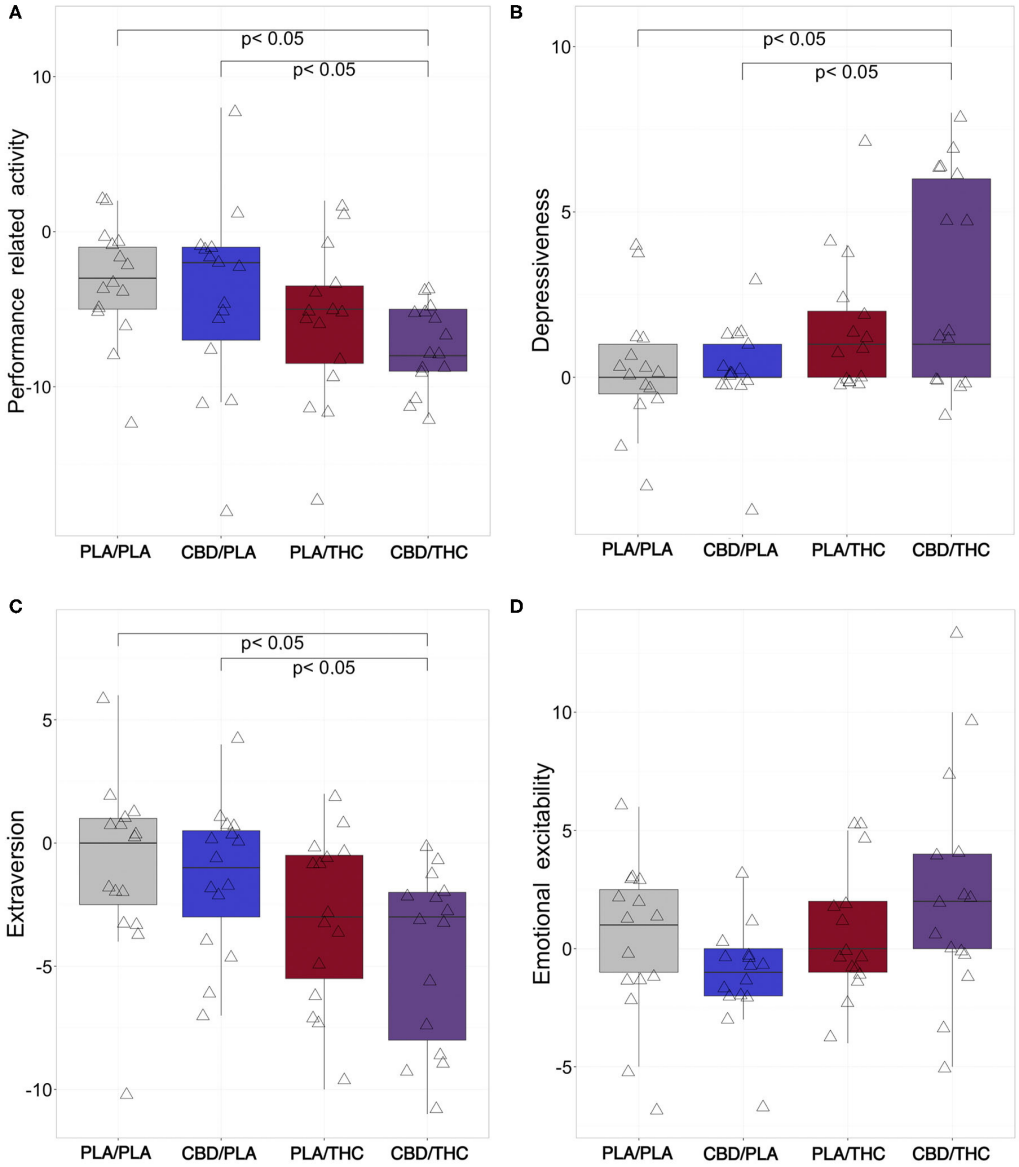
Changes of aspects of emotion from baseline to investigational assessment. From six emotional categories covered by the adjective mood rating scale (EWL), four categories were differentially affected by cannabinoid treatment. **(A)** Subjects who received the 800 mg of cannabidiol orally (CBD) prior to 20 mg of delta-9-tetrahydrocannabinol orally (THC; CBD/THC) showed significantly reduced performance-related activity difference scores (investigational day—baseline) in comparison to placebo (PLA) prior to placebo (PLA/PLA) (*p* = 0.002), and CBD prior to placebo (CBD/PLA) (*p* = 0.035) treated participants. Furthermore, the administration of CBD followed by THC (CBD/THC) led to significantly more pronounced self-rating of depressiveness compared to PLA/PLA (*p* = 0.015) and CBD/PLA (*p* = 0.026) at the investigational day **(B)**. Rating of extraversion **(C)** was significantly lower in subjects treated with CBD/THC in comparison to PLA/PLA (*p* = 0.013) and CBD/PLA (*p* = 0.017), indicating a more introverted behavior. **(D)** Regarding emotional excitability, the overall group difference did not reach significance (*p* = 0.058). However, by trend, participants treated with CBD/PLA showed decreased excitability difference scores, while the administration of THC/CBD resulted in increased values. PLA/PLA, placebo/placebo; CBD/PLA, cannabidiol/placebo; THC/PLA, delta-9-tetrahydrocannabinol/placebo; CBD/THC, cannabidiol/ delta-9-tetrahydrocannabinol.

All subjects showed slightly reduced performance-related activity compared to baseline. Interestingly, subjects who received CBD/THC showed significantly lower performance-related activity difference scores (mdn = −8, −12, −9, −5, −4 [median, percentiles 0, 25, 75, 100]) compared to PLA/PLA (mdn = −3, −12, −5, −1, 2, *p* = 0.002) or CBD/PLA (mdn = −2, −18, −7, −1,8, *p* = 0.035, see Figure 2A). The change in performance-related activity was solely lower by trend in CBD/THC. The difference score did not differ from those observed in the PLA/THC group but from those of the PLA/PLA and CBD/PLA group.

In the category depressiveness, CBD/PLA subjects showed similar scores compared to baseline. PLA/THC and CBD/THC led to higher depressiveness scores compared to baseline, although significance was only reached in CBD/THC subjects (mdn = 1, −1, 0, 6, 8) vs. PLA/PLA (mdn = 0, 4, 0, 1, 3, *p* = 0.015) and vs. CBD/PLA (mdn = 0, −4, 0, 1, 3, *p* = 0.026, Figure 2B).

A similar pattern was observed for the emotional category extraversion. While after PLA/PLA and CBD/PLA treatment the extraversion scores were comparable to baseline, PLA/THC and PLA/THC treatment resulted in a slight decrease of extraversion, representing a more introverted behavior in this group. However, only CBD/THC treatment led to a significant decline in extraversion scores (mdn = −3, −11, −8, −2, 0) vs. PLA/PLA (mdn = 0, −10, −2.5, 1, 6, *p* = 0.013) and vs. CBD/PLA (mdn = −1, −7, −3, 0.5, 4, *p* = 0.017, Figure 2C).

Regarding emotional excitability, the overall group difference did not reach significance (Kruskal-Wallis: *p* = 0.058). Nevertheless, by trend emotional irritability seemed to be lower after CBD/PLA treatment, while subjects who took CBD/THC showed increased excitability (Figure 2D).

### Effects of Exogenous Cannabinoids THC and CBD on Cognition and Attention

**Figure 3:**
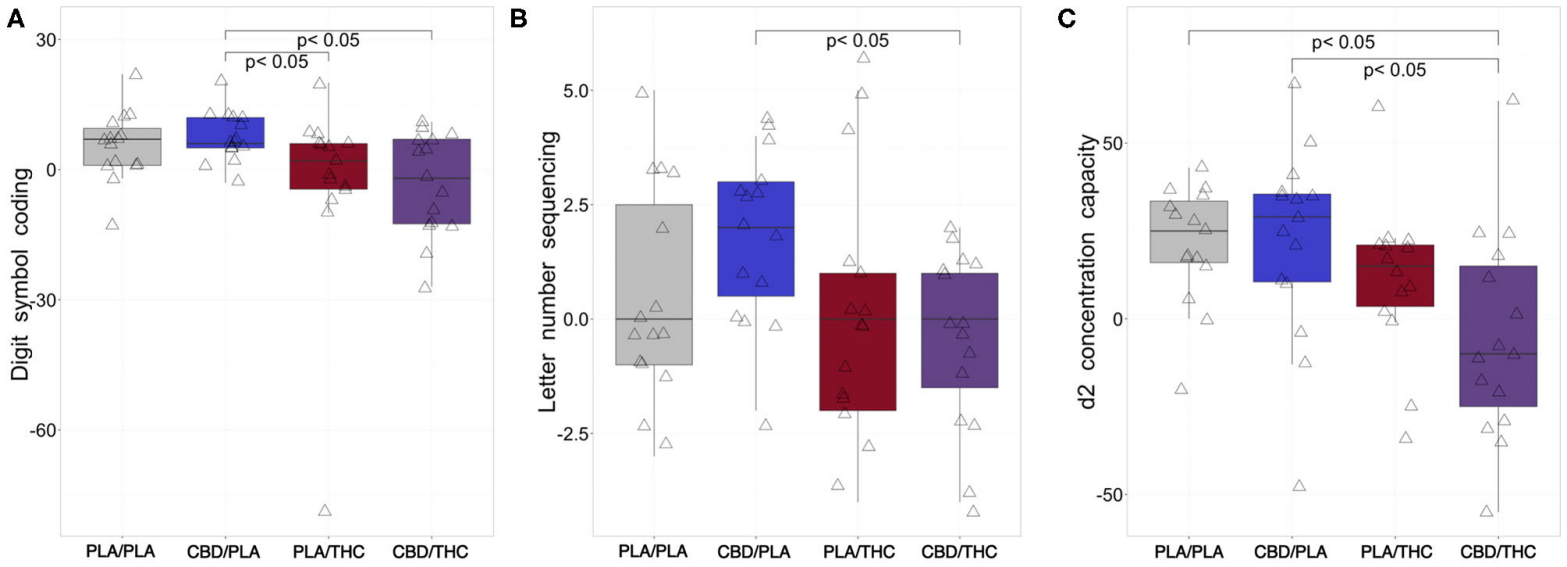
Changes of cognition from baseline to investigational assessment. **(A)** While subjects treated with placebo (PLA) prior to placebo (PLA/PLA) and 800 mg cannabidiol orally (CBD) prior to placebo (CBD/PLA) improved their cognitive processing at the interventional day compared to baseline (reflected by positive difference scores in the Digit Symbol Coding task), subjects receiving 20 mg of delta-9-tetrahydrocannabinol orally (THC) proceeded by placebo (PLA/THC) or CBD (CBD/THC) showed a slightly reduced performance. The difference scores of both PLA/THC and CBD/THC) was significantly lower compared to the CBD/PLA group (PLA/THC: *p* = 0.039; CBD/THC: *p* = 0.016). **(B)** After CBD/PLA, the change in working memory performance (from baseline to 205 min post drug intake) assessed by the Letter Number Sequencing task seemed to be most pronounced. Interestingly the difference score observed for CBD/PLA was significantly higher than the score of the CBD/THC group (*p* = 0.005). **(C)** At the interventional day, we observed higher mean attentional d2 scores compared to baseline after PLA/PLA, CBD/PLA, and PLA/THC treatment. In subjects who received CBD/THC, an increased attentional performance was not observed, and the difference score was significantly lower compared to the PLA/PLA (*p* = 0.005) and CBD/THC (*p* = 0.010) group. PLA/PLA, placebo/placebo; CBD/PLA, cannabidiol/placebo; THC/PLA, delta-9-tetrahydrocannabinol/placebo; CBD/THC, cannabidiol/ delta-9-tetrahydrocannabinol.

Compared to the first assessment at the baseline visit (V1), subjects receiving PLA/PLA or CBD/PLA showed increased performance in both cognitive tests at the interventional day (Figures 3A,B).

On the other hand, negative digit symbol coding difference scores, indicating impaired cognitive processing, were found after PLA/THC and CBD/THC administration. However, this reduction was only significant in comparison of CBD/PLA (mdn = 6, −3, 5, 12, 20) vs. PLA/THC (mdn = 2, −79, −4.5, 6, 20, *p* = 0.039) and vs. CBD/THC (mdn = −2, −27, −12.5, 7, 11, *p* = 0.016; Figure 3A).

In addition, the working memory performance assessed by the Letter Number Sequencing test was significantly lower in the group receiving CBD/THC (mdn = 0, −4, −1.5, 1, 2) compared to CBD/PLA (mdn = 2, −2, 0.5, 3, 4, *p* = 0.005; Figure 3B).

The d2 Test of Attention was applied three times within 3 days and mean scores averaged over all participants increased continuously. Information processing speed displayed in this test by the error corrected total number (32) augmented from V1 (mdn = 536, 293, 461, 556.25, 618.00) over V2 (mdn = 539, 329.0, 456.5, 583.0, 652) 523.8 to V3 (mdn = 593.5, 414.0, 547, 639.5.653).

Like in the cognitive tests, we observed positive difference scores of concentration capacity in participants treated with PLA/PLA or CBD/PLA, indicating better test results at the interventional day compared to baseline testing. In addition, participants of the PLA/THC group showed higher concentration capacities at V2 than at baseline testing, while subjects receiving CBD/THC were less focused, indicated by negative difference scores. The reduction of attention after CBD/THC administration (mdn = −10, −55, −25, 15, 62) was significant compared to PLA/PLA (25, −20, 16, 33.5, 43, *p* = 0.005) and CBD/PLA treatment (mdn = 29, −48, 10.5, 35.5, 67, *p* = 0.010; Figure 3C).

### THC Blood Levels

Subjects who received PLA/THC showed slightly lower THC blood levels (mdn = 0.954, 0.0, 0.817, 1.615, 9.690 pmol/ml) compared to subjects who received CBD/THC (mdn = 2.61, 0.614, 1.032, 3.960, 8.350 pmol/ml). However, this difference did not reach statistical significance.

## Discussion

In the current study, we investigated the effects of a single oral PLA/PLA, CBD/PLA, PLA/THC, and CBD/THC time-shifted double-dummy administration (CBD or corresponding PLA prior to THC or corresponding PLA treatment) on cognitive processing speed, working memory, attention, and emotion in healthy volunteers. Although previous reports suggested that CBD and THC interact on at least some cognitive domains and anxiety, a single oral dose CBD, administered prior to THC, was insufficient to mitigate the detrimental effects of THC in our setting.

### Effects of CBD and THC on Emotion

We observed no significant influence of PLA/PLA and CBD/PLA treatment on the six emotional categories (a) performance-related activity, (b) general inactivation, (c) extroversion, (d) general well-being, (e) emotional excitability, and (f) depressiveness. Slight reductions in performance-related activity in both groups could be most likely traced back to the intense investigational day. However, CBD/PLA might favor a slightly more relaxed emotional state compared to PLA/PLA, as the mean difference scores in emotional excitability were lower by trend. These findings are in line with a recent study, showing that a single CBD administration did not affect the three main dimensions of affect (hedonic tone, energetic arousal, and tense arousal) assessed by the University of Wales Mood Adjective Checklist (UMACL) (20). However, it has also been reported that subjects felt more quick-witted and clear-minded (18) after acute CBD treatment, while another study found increased Adjective Mood Rating Scale (Bf-S) scores, indicating a decline in general well-being, 3 h after CBD treatment (19). Hitherto, available literature on the emotional effects of CBD in humans focuses mainly on anxiolytic properties. Results of published studies indicate that CBD does not affect anxiety *per se*, as it has no effect on baseline anxiety (18, 19, 35, 36). Our results are consistent with these findings in the most general sense, although the used adjective mood rating scale does not retrieve anxiety, but depressiveness experience. However, it has been reported that CBD exhibits anxiolytic properties in experimental anxiogenic settings (18, 37) and seems to be effective in subjects with social phobia and generalized social anxiety disorder (38, 39). Recreational use of cannabis is mostly associated with feeling “high” and relaxing properties (40), while only a subset of naïve users reports dysphoria (41) and even paranoia (40). In the present study, we observed that THC administration reduced performance-related activity and extraversion by trend, while subjects felt slightly more depressive compared to PLA/PLA and CBD/PLA treated subjects. These results are consistent with previous studies, reporting that healthy volunteers acutely treated with THC feel anxious (18) and tense (35). Acute cannabis use has also been associated with an euphoriant effect (“high”), decreased anxiety, depression, and tension, as well as increased sociability if taken in friendly surroundings (40). This seems contradictory, but in line with a proposed biphasic effect of THC. While low doses [around 5–10 mg THC- in a joint (40)] have revealed anxiolytic effects, higher doses can induce dysphoric experiences (40).

As mentioned above, CBD may reduce experimentally induced anxiety. It has been shown that CBD also reverses the acute anxiogenic effects of THC when both cannabinoids were administered simultaneously (18, 35). Thus, we hypothesized that CBD pre-treatment might alleviate THC effects on depressiveness and extraversion. Surprisingly, the administration of THC subsequent to a single dose of CBD had nearly the same effect on emotional perception as the THC treatment following placebo, as reflected by significantly higher depressiveness and significantly lower extraversion scores. This finding is indicative of more complex pharmacodynamic interaction of THC and CBD, depending on the treatment regime and eventually, the dosages, and consistent with the observation that different compositions of THC and CBD content in medicinal cannabis influence anxiety, depression, and stress in different ways (42). Noteworthy, Cuttler et al. (42) also suggested a difference in acute and long-term effects, whereby initially reduced perceived symptoms of negative affect may exacerbate baseline symptoms of depression over time, further demonstrating the complexity of the phenomenon.

### Effects of CBD and THC on Cognition and Attention

We observed that participants treated with PLA/PLA scored higher at the investigational day in both cognitive tasks and the d2 Test of Attention. This improvement might be due to learning- and training effects, even though the tasks were repeated in different versions for repeated measures. Similar results were observed for subjects treated with CBD/PLA, indicating that CBD did negatively influence cognitive processing speed and attention, although ameliorating working memory performance by trend.

PLA/THC administration resulted in reduced cognitive processing speed compared to CBD/PLA treatment, although the performance remained widely unaffected compared to baseline. This difference to CBD/PLA is likely due to the improvement of the latter group on the investigational day, also seen in PLA/PLA. Therefore, it may be speculated if THC reduced a potential learning effect or other factors are contributing to this finding. However, the observed group differences do probably not reflect difficulties in accessing working memory, which remained largely unaffected or long-term memory contents, as Ranganathan and D'Souza (43) showed that THC does not disrupt access to information learned before administration.

Subjects treated with PLA/THC or CBD/THC reported reduced subjective performance-related activity, with CBD/THC even becoming significant vs. PLA/PLA and CBD/PLA as another indicator of more pronounced THC effects in the CBD/THC group, potentially related to the reported higher THC plasma levels. This subjective reduction in performance-related activity is unlikely attributed to the intense investigational day, as this reduction was not apparent in the PLA/PLA and CBD/PLA groups. While a reduced subjective performance-related activity was reflected by the impairments in cognitive processing speed (Digit Symbol Coding Task) and concentration capacity (d2 Test of attention), a discrepancy between subjective experience and objective performance is noteworthy and has been observed before (44).

Interestingly, THC did not influence working memory performance. This finding is consistent with a study reporting that THC did not alter working memory whilst disturbing episodic memory and verbal learning (45). Unfortunately, the latter domains were not assessed by the tasks applied in the present study. However, other studies reported that intravenous as well as pulmonal THC administration induced working memory impairments, reflected by reduced performance in the Digit Span forward and reverse task (20), the Digit Symbol Substitution Task (46, 47), the Paced Auditory Serial Addition Task and the spatial N-back task (48).

Although subjects treated with PLA/THC still showed higher attentional scores than at baseline, this increase was less pronounced compared to PLA/PLA and CBD/PLA treatment. Again, it might be that learning effects superimpose impairments provoked by THC, as it has been previously reported that THC treatment resulted in a significant decrease of d2 test performance compared to placebo (49) and Divided Attention Task performance compared to baseline assessment (47). Furthermore, the synthetic THC analog nabilone led to a dose-dependent deterioration of attention in the Cognitive Drug Research computerized assessment system (50).

As already discussed with regard to emotional states, we hypothesized that CBD is able to counteract the effects of THC on cognition and attention. Interestingly, this was not the case in our setting. Subjects who received CBD/THC treatment showed no improvement in cognitive processing speed, working memory, and attention compared to subjects who received PLA/THC. Probably based on the slightly higher THC levels in the CBD/THC group, the effects of THC were more pronounced. We observed significantly reduced cognitive processing speed, working memory, and attention compared to CBD/PLA and PLA/PLA. At first, this is surprising, as it has been shown that CBD pre-treatment (600 mg orally) is able to diminish impairments of episodic memory induced by intravenously injected THC (20). However, the same study reported that CBD pre-treatment did not attenuate THC induced impairments on immediate recall, digit-span forward, and digit span backward (20). Furthermore, vaporized THC (8 mg) alone and combined with vaporized CBD (16 mg) showed the same level of impairment in episodic memory on prose recall in the story recall task from the Rivermead Behavioral Memory Test when compared to placebo (48). Interestingly, acute treatment with CBD (300 or 600 mg) did also not improve the performance of schizophrenia patients in the Stroop Color Word Test (51), and thus did not affect selective attention and processing speed in patients. It may well be that CBD pre-treatment is more effective on other cognitive domains than those applied in the present study, as it had been suggested that CBD, in particular, protects hippocampal-dependent memory performance from the impact of THC (20). On the other hand, it might also be that repeated CBD treatment is required to activate mechanisms, improving cognitive and attentional performance while acute CBD administration is not sufficient. However, Arkell et al. (46) observed a reduction of attention after a combined CBD/THC administration in the Divided Attention Task, while the task performance was not affected by THC alone.

### Limitations to Our Study

Although we tried to address several issues affecting studies on THC and CBD in healthy volunteers (52), there are still a number of limitations to this study.

First, we only recruited male subjects due to the initially planned parallel study using radionuclides to investigate cannabinoid receptor availability after cannabinoid intake. However, due to regulatory changes, we had to halt this study indefinitely shortly before the initiation of our trial reported here. Thus, our data lacks generalisability with regard to gender.

Second, we observed substantial interindividual differences. The two major approaches to minimize standard deviations are increasing the number of subjects and homogenizing the sample cohort. The number of 60 participants was already higher than in the majority of comparable studies. Furthermore, our study was designed to reduce sample heterogeneity by stratifying for functional COMT polymorphism to exclude the influence of different dopamine elimination rates, as it has already been shown that cannabis affected cognition in a COMT genotype-dependent manner (27, 28).

Third, we included only subjects with body mass indices ranging from 18 to 30 and provided equivalent meals for all participants throughout the interventional day to achieve a comparable cannabinoid uptake. However, providing individual weight adapted dosing would be even better, but this was not feasible due to limitations of differential dosing per os. Further, oral administration of drugs is accompanied by a delayed digestion-dependent uptake and variable metabolization in the liver due to individual enzyme activities. Inhalative and intravenous applications of cannabinoids bypass the first-pass effect in the liver and result in faster peaks (43, 53), and may also reduce interindividual differences. However, oral administration leads to more consistent long-lasting peak concentrations and is also used in other clinical trials. They represent a route of administration more suitable for medical use. In particular, the latter is essential as CBD has recently been approved as an orphan drug for Dravet and Lennox-Gastault syndrome in children and is currently investigated regarding its beneficial effects in other diseases, e.g., schizophrenia. We have recently demonstrated in rodents that different pharmaceutical preparations of THC can influence its behavioral effects depending on the kinetics of the surge of THC (54), suggesting that the investigation of both oral as well as inhalative/intravenous administration of THC and CBD is justified.

Fourth, we investigated the change from baseline to post-drug intake to reduce general interindividual differences. However, multiple testing resulted in potential learning effects in both cognitive tasks and attention testing, even when using appropriate test/re-test paradigms. These learning effects might have superimposed some cannabinoid effects on cognition and attention.

Fifth, the potential use of caffeine, nicotine, and alcohol has been out of our rigid control between baseline (V1) and the following investigational day as subjects were not kept in a closed environment such as an experimental ward for compliance and funding reasons. However, subjects were thoroughly screened for any history of use or even abuse of these compounds and did neither report more pronounced use nor demonstrate any clinical signs related to it, particularly not during visits or the extensive time they were under direct observation during the trial.

Sixth, we tested only one dose of CBD and THC, respectively. The THC dose was chosen based on the recommended maximum daily dosage of dronabinol and the available oral dosage forms, while the dose of CBD was based on the dosage used in our previous clinical trial in schizophrenia (55), demonstrating the antipsychotic effects of CBD. However, it may be that the effects induced by the high THC dose were too strong to be controlled by CBD. Thus, future studies should investigate the effects of a broader range of CBD on various THC doses.

## Conclusions

This study showed that CBD has no detrimental effects on emotion, cognition, and attention. However, our results do not provide further evidence that acute CBD administration improves impairments of cognitive functions and attention or alteration of emotional experience induced by THC in healthy volunteers under any circumstances.

As there is some evidence that CBD may be effective with regard to other cognitive domains than those investigated in the present study, further studies are needed to elucidate the intricate interrelation of both phytocannabinoids and cognition. Further, the effects of repeated or longer-term CBD administration should be investigated, particularly with regard to the increasing medical interest in CBD as an (investigational) medical drug.

## Data Availability Statement

The datasets presented in this article are not readily available because Data belongs to the sponsor of the clinical trial (Central Institute of Mental Health) and requires previous consent of the sponsor. Requests to access the datasets should be directed to markus.leweke@sydney.edu.au.

## Ethics Statement

The studies involving human participants were reviewed and approved by Ethics Committee II of the Medical Faculty Mannheim, Heidelberg University, Germany. The participants provided their written informed consent to participate in this study.

## Author Contributions

FML and CR conceived and designed the experiments with input from DK, MH, BL, JKM, and AR. FML, JM, BL, AR, CR, AMS, and TW performed the experiments. TW and CR analyzed the data with input from FML and MH. TW and CR drafted the manuscript with input from MH and FML. All authors contributed to final manuscript preparation, read, and approved the final manuscript.

## Conflict of Interest

FML is a shareholder of curantis UG (ltd). The remaining authors declare that the research was conducted in the absence of any commercial or financial relationships that could be construed as a potential conflict of interest.
